# Nurses’ knowledge and attitudes regarding children’s pain assessment and management in Nepal

**DOI:** 10.1177/13674935231195133

**Published:** 2023-08-11

**Authors:** Jagamaya Shrestha-Ranjit, Uma Devi Ranjitkar, Tineke Water, Sulochana Shrestha, Chandrakala Sharma, Suzanna Mukhia, Jamuna Adhikari, Tulashi Adhikari, Archana Pandey, Muna Sharma, Apsara Pandey, Nibaran Joshi, Natalie Tuck

**Affiliations:** 1 Faculty of Health and Environmental Sciences, 1410 Auckland University of Technology, Auckland, New Zealand; 2 198814 Institute of Medicine Maharajgunj Nursing Campus, Tribhuvan University, Kathmandu, Nepal; 3 527323 Tribhuvan University Institute of Medicine Birgunj Nursing Campus, Birgunj, Nepal; 4 Kanti Children’s Hospital, Kathmandu, Nepal; 5 Waitematā Pain Services, Department of Anaesthesia and Perioperative Medicine, Te Whatu Ora Waitematā

**Keywords:** Child, infant, pain measurement, pain management, nursing knowledge, Nepal, low- and middle-income countries, paediatric nursing, education

## Abstract

Pain is frequently experienced by children in hospital, and international guidelines for appropriate pain assessment and management are available. Optimal management of paediatric pain has important long-term health, psychosocial, and economic benefits. However, evidence indicates that globally there are deficits in nurses’ understanding of paediatric pain assessment and management. This study explored knowledge and attitudes regarding paediatric pain assessment and management among nurses at a tertiary children’s hospital in Nepal. In this cross-sectional study all 140 nurses at a tertiary children’s hospital in Nepal, were invited to complete the validated Paediatric Nurses Knowledge and Attitudes Survey. Findings revealed substantial deficits in nurse’s knowledge and erroneous attitudes towards pain assessment and management in children. Test scores ranged from 14% to 56%, with mean scores of 38%, with no nurses achieving a recommended pass score of 80% regarding knowledge and attitudes in paediatric pain management. Consistent with previous research, nurses had insufficient knowledge and attitudes that did not reflect best practice regarding pain assessment and management in children. Education programmes targeting both trainees and registered nurses are essential to enable nurses to deliver evidence-based care and improve outcomes for children and their families.

## Introduction

Within hospital settings children frequently experience acute pain due to injury, illness, or disability (Friedrichsdorf and Goubert, 2020). Additionally, pain is frequently caused by medical treatment itself, such as painful diagnostic, investigative, or therapeutic procedures (Friedrichsdorf and Goubert, 2020). Poorly managed paediatric pain is associated with detrimental changes in pain processing and perception (Schwaller and Fitzgerald, 2014) and negatively affects social, neurocognitive, and physiological outcomes (Friedrichsdorf and Goubert, 2020). Conversely, effective pain management reduces time spent in hospital, enhances healing and recovery, facilitates early mobilisation, and reduces suffering and healthcare costs (Stevens et al., 2014). Access to appropriate pain management is considered a fundamental human right (International Association for the Study of Pain, 2014), and practice guidelines include pain assessment as the ‘fifth vital sign’ with self-report accepted as the gold standard for pain assessment (American Academy of Pediatrics, 2001). However, despite the importance of optimal pain management, and international guidelines for the assessment and treatment of pain in children (International Association for the Study of Pain, 2014, World Health Organisation, 2020), paediatric pain remains poorly managed in hospital settings (Friedrichsdorf and Goubert, 2020, Twycross et al., 2016, Vejzovic et al., 2020).

Accurate assessment of paediatric pain is essential to guide pain management interventions. Various paediatric pain assessment tools have been developed and recommended to use. However, paediatric pain assessment tools are reported be under-utilised (Carter et al., 2016). Several factors have been identified as contributing to inadequate pain assessment and management in children (Alotaibi et al., 2018, Whitley et al., 2021). In particular, although the IASP has published discipline specific pain teaching curricula, including for nurses (International Association for the Study of Pain, 2017), medical training facilities do not provide sufficient education regarding pain mechanisms, assessment, and management (Mackintosh-Franklin, 2017). Additionally, attitudes regarding necessity or feasibility of pain management differ across cultures (Lovering, 2006), and paediatric pain management is complicated by factors such as developmental stage, parental beliefs, and language proficiency; and children who may have difficulty communicating their level of pain or the effectiveness of analgesics to adults (Alotaibi et al., 2018). Given these difficulties, nurses responsible for pain assessment and treatment in children must have a high level of knowledge, and appropriate attitudes regarding pain and its management in order to deliver safe and effective care (Alotaibi et al., 2018). However, numerous studies have documented substantial deficits in nurses understanding of paediatric pain and its management (Alotaibi et al., 2018, Mediani et al., 2017, Ung et al., 2016).

Most work examining paediatric nurses’ understanding of pain has used the Pediatric Nurses Knowledge and Attitudes Survey (PNKAS) (Manworren, 2001). The PNKAS assesses knowledge and attitudes relating to analgesic administration and duration, beliefs about opioid addiction, pain assessment, calculating medication doses, and administering medications. The PNKAS was developed to reflect international guidelines regarding pain assessment and treatment and was updated in 2014 (Ferrell and McCaffery, 2014). Although it has been suggested that scores over 80% on the PNKAS indicate the ability to deliver safe and effective nursing care to children with pain (McCaffery and Robinson, 2002), studies have repeatedly found that both nursing students and registered nurses score well below this level.

Nepal is a landlocked South Asian country bordered by India and China. Nepal has a population of 29.14 million, with 20% living in urban centres and the remaining 80% in rural settings (United Nations Development Programme, 2020). Nepal has a young population, with a median age of 24.6 years, and a life expectancy of 70.8 years. Nepal has a Human Development Index (HDI) of 0.602 placing it in the ‘medium’ category for human development factors, alongside countries such as Ghana, Vanuatu, Timor-Leste, Kenya, and Cambodia (United Nations Development Programme, 2020). Prior to 2019, Nepal was classified by the World Bank as a low-income country (LIC), but has recently been re-classified as lower middle income (LMIC) (The World Bank Group, 2017). Malnutrition remains high, with 36% of children under five experiencing moderate-severe malnutrition, and 25% of the population living below the national poverty line (United Nations Development Programme, 2020).

Given competing health concerns, pain is not a recognised research priority in Nepal, and is not included in the Nepal Health Research Council’s (NHRC) research priority agenda (Nepal Health Research Council, 2019). In 2012, the Essential Pain Management (EPM) education programme, specifically designed for low-middle income countries, was introduced to Nepal (Goucke et al., 2015, Marun et al., 2020) and was pivotal in establishing the Nepal Association for the Study of Pain (NASP) in 2017 (Subedi et al., 2020). Prior to 2018 only 19% of health professionals at five tertiary hospitals reported having received pain management training and only 40% consistently used pain scales for pain assessment (Shakya et al., 2020). By 2018, 15 EPM workshops had been delivered to over 700 participants in Nepal, and those facilitating the programme describe a strong positive response, including an increase in clinicians training in advanced pain management, improvement in the assessment of pain, and one institute making EPM training compulsory for all first-year resident doctors (Subedi et al., 2020).

Two studies in Nepal have assessed pain knowledge and attitudes among nurses in adult services. In one study undertaken in 2016, 85 nurses from a single hospital completed the modified Nurses Knowledge and Attitudes Survey Regarding Pain (NKAS). Mean scores were 37%, with most participants scoring below 50%. There was no statistically significant relationship between knowledge and attitudes and characteristics such as age, education, or area of work, however previous experience in pain management was associated with higher scores (Shakya and Shakya, 2016). A study in 2020 looked at knowledge, attitudes, and practices of nurses in post-operative wards across four hospitals in Nepal, with findings indicating that 85% of nurses had a low level of knowledge (Thapa and Gurung, 2020). Although these studies suggest that knowledge and attitudes regarding pain in Nepal do not meet international standards for evidence-based practice, no studies have examined pain-related knowledge and attitudes of nurses in paediatric settings. Given substantial long-term social, economic, psychological, and health-related costs of poorly managed paediatric pain (Schwaller and Fitzgerald, 2014), and evidence of poor knowledge and attitudes regarding pain among paediatric nurses globally; this study was designed to assess nurses’ knowledge and attitudes regarding paediatric pain assessment and management at a tertiary paediatric hospital in Nepal.

### Aim

To explore nurses’ knowledge and attitudes regarding paediatric pain assessment and management at a tertiary paediatric hospital in Nepal.

## Methods

### Study design and setting

For this cross-sectional survey, data were collected at a tertiary Children’s Hospital in Nepal. This is the only paediatric tertiary hospital in Nepal, which is managed by the Hospital Development Board under the Ministry of Health. This hospital currently has 300 beds, and it has planned to add 200 more beds.

### Ethical consideration

Ethics approval was obtained from the University Ethics Committee (Ref: 19/298), the Institutional Review Committee of the Hospital (Ref: 511), and the Nepal Health Research Council (Ref: 1605).

## Participants

Nurses were eligible to participate in this study if they were working at the hospital as registered nurses for a minimum of 6 months prior to the start of the study. The nurses who have been working at the hospital for less than 6 months were excluded from participating in this study as they might have had very limited experience regarding assessment and management of pain in paediatric setting. All 140 registered nurses working at the hospital were invited to complete an anonymous paper-based survey over a 3-week period (17 January–5 February 2021). Surveys and participant information sheets were placed in communal areas in all hospital wards, and a sealed collection box was available in staff rooms of each ward for nurses to drop off completed anonymous surveys.

### Data collection

Sample characteristics including age, nursing experience, academic qualifications, and the hospital department worked in were collected.

The validated Pediatric Nurses Knowledge and Attitudes Survey Regarding Pain (PNKAS) (Manworren, 2001) measures knowledge and attitudes regarding pain assessment, general pain management, risk of opioid addiction, and use of analgesics. It is based on Ferrell and McCaffery’s Nurses’ Knowledge and Attitudes Regarding Pain (NKAS) questionnaire (McCaffery, 1997) and has been adjusted for use among paediatric nurses (Manworren, 2001). The PNKAS is a 42-item survey comprising 25 binary (true/false) items, 13 multiple choice questions, and four items relating to two case study vignettes. Correctly answered items are scored ‘1’ and incorrectly answered items are scored ‘0’ with total scores ranging from 0 to 42 which are then converted to a percentage ([total/42]*100) with higher scores indexing greater number of correct items (Manworren, 2001). The PNKAS has acceptable stability (test-retest reliability = 0.67), and adequate internal reliability (Cronbach’s alpha of 0.72–0.82) (Manworren, 2001). Content of the PNKAS was developed based on standards and guidelines by the Agency for Healthcare Policy and Research, the American Pain Society, and the World Health Organization (Manworren, 2001). The PNKAS has been translated into multiple languages and used across diverse healthcare settings. For the present study, the English version of PNKAS was used to gather data. The PNKAS does not differentiate between knowledge and attitudes, rather it is recommended that data are analysed to identify items with the fewest correct responses and the items that are answered well in order to guide educational needs (Ferrell and McCaffery, 2014). As with prior work, a total score of 80% was considered indicative of satisfactory knowledge and attitudes regarding children’s pain (Ung et al., 2016). In the present study, Cronbach’s alpha was α = 0.82 indicating good internal consistency.

### Statistical analyses

Statistical analyses were performed with SPSS Statistics version 27 (IBM Corporation, New York). Descriptive statistics were used to examine sample characteristics and PNKAS scores. Categorical variables are presented as frequencies and percentages, and continuous variables are reported as means and standard deviations (SD). The single outcome measure, test scores, was screened for normality by examining histograms and estimates of skewness and kurtosis. No deviations from normality were evident, with the Shaprio–Wilk test supporting normally distributed data; W (110) = 9.79, *p* =.079. The homogeneity of variance and independence assumptions were met; therefore, one-way ANOVA models were used to identify whether PNKAS test scores differed across groups, and differences in PNKAS scores between any two groups were examined using two-tailed independent samples *t* test. Statistical significance for all tests was set at the level of α = 0.05.

## Results

Of a total pool of 140 potential participants who were invited to participate in the study, 116 surveys were returned. Three participants, who did not answer any of the questions, and three participants, who answered fewer than 12/42 questions, were excluded from analyses. The remaining 110 participants, who correctly answered questions were scored ‘1’ with incorrect or unanswered questions scored as ‘0’.

### Demographics and sample characteristics

The mean age was 31.7 (SD = 9.1) years. The highest level of education attained by participants was a master’s degree in nursing (n = 4, 3.6%), followed by a bachelor’s degree in nursing (*n* = 76, 69.1%) and a proficiency certificate level in nursing (*n* = 30, 27.3%). Participants had a mean of 9.5 (SD = 0.8) years of experience. See Table 1.

**Table 1.** Sample demographics and ANOVA tests for between group differences in PNKAS test scores.

	Valid N	N	%	Mean (SD)	ES	95% CI	Omnibus test statistic
Age in years	110						
20–25		32	29.1	37.65 (7.44)	0.00	0.00,0.00	F = 0.01
26–32		41	37.3	37.49 (8.67)			
33+		37	33.6	37.46 (8.13)			
Years nursing experience	107						
0–3		34	30.9	36.73 (7.46)	0.01	0.00, 0.05	F = 0.38
4–10		38	34.5	38.24 (8.37)			
11+		35	31.8	36.94 (8.42)			
Academic qualification (N, %)	110						
*PCL Nursing*		30	27.3%	36.27 (6.57)	0.05	0.00, 0.13	F = 2.60
*BSN/BNS/PBBN*		76	69.1%	37.58 (8.36)			
*MN/MSN*		4	3.6%	45.93 (9.75)			
Pain specific training (N/%)	110						
*Yes*		7	6.4%	40.86 (5.83)	0.44	−0.33, 1.21	*t* = 1.13
*No*		103	93.6%	37.30 (8.17)			
Specific protocol in pain management	109						
*Yes*		31	28.2%	35.41 (7.96)	0.36	−.78, 0.06	*t* = 1.69
*No*		78	70.9%	38.28 (8.04)			
Department	105						
*ED*		15	14.3	33.33 (9,12)	0.129	0.00, 0.19	F = 1.77
*Surgical*		17	16.2	36.11 (6.84)			
*Special (Paying) Cabin*		9	8.6	35.14 (6.62)			
*PICU*		21	20.0	37.43 (7.99)			
*NICU*		7	6.7	36.88 (10.86)			
*HDU*		10	9.5	38.14 (7.84)			
*OPD*		4	3.8	40.12 (3.49)			
*Oncology*		11	10.5	42.92 (8.66)			
*Medical*		11	10.5	41.86 (7.50)			

Valid *N* = number of participants with complete data; n = subset of the total respondents; M = mean; SD = standard deviation, ES = effect sizes reported as Eta-squared for the F statistic or Cohen’s D for t tests, CI = confidence interval of the ES, PCL = proficiency certificate level in nursing; BSN/BNS/PBBN = bachelor’s degree in nursing, MN/MSN = master’s degree in nursing, ED = emergency department, Special (Paying) Cabin = private hospital care; PICU = paediatric intensive care unit; NICU = neonatal intensive care unit; HDU = high dependency unit; OPD = outpatient department

### Knowledge and attitudes (PNKAS scores)

The mean total score of correctly answered items was 37.53% (SD = 8.07%). Scores ranged from 14% to 56%. Items answered correctly by most nurses (≥70%) were items 5, 22, 8, 27, and 30. Specifically, most nurses knew that children/adolescents can experience different levels of pain from similar stimuli (item 5, correctly answered by *n* = 90/82%), and that following an initial dose of opioid analgesic, subsequent doses should be adjusted according to the patient’s response (item 22, correctly answered by *n* = 86/78%). Most nurses also knew that children undergoing repeated painful procedures should receive maximum analgesics at the first procedure, to minimise anxiety related to future procedures (item 8, correctly answered by *n* = 83/76%), and that opioid administration for sudden, severe, acute pain should be delivered intravenously (item 27, correctly answered by *n* = 79/72%).

Survey items most often scored incorrectly were items; 38, 7, 40a, 39a, and 39b (see Table 2). Specifically, nurses overestimated the risk of addiction when treating pain with opioid analgesics (item 38, correctly answered by *n* = 7/6%), and very few nurses knew that non-pharmacological interventions can be helpful for severe pain (item 7, correctly answered by *n* = 5/6%). Most nurses indicated that they would not administer the most appropriate dose of morphine in a given patient scenario (item 39b, 5% correct), and nearly all nurses underestimated an adolescent’s pain intensity in two patient scenarios (item 40a, 2% correct, and item 39a, 0% correct) See Table 2.

**Table 2.** Questionnaire items ordered (highest to lowest) according to the percentage of nurses scoring the item correctly.

Item	Question	*n*	%
5	Comparable stimuli in different people produce the same intensity of pain	90	81.8
22	After the initial recommended dose of opioid analgesic, subsequent doses should be adjusted in accordance with the individual patient’s response	86	78.2
8	Children who will require repeated painful procedures (e.g., daily blood draws), should receive maximum treatment for the pain and anxiety of the first procedure to minimise the development of anticipatory anxiety before subsequent procedures	83	75.5
27	The recommended route of administration of opioid analgesics to children with brief, severe pain of sudden onset, for example, trauma or post-operative pain is intravenously	79	71.8
30	Analgesics for post-operative pain should initially be given on a fixed schedule	77	70.0
28	Which of the following analgesic medications is considered the drug of choice for the treatment of prolonged moderate to severe pain for children with cancer?	70	63.6
18	The child/adolescent with pain should be encouraged to endure as much pain as possible before resorting to a pain relief measure	66	60.0
32	Analgesia for chronic cancer pain should be given	65	59.1
14	Parents should not be present during painful procedures	63	57.3
17	Young infants, less than 6 months of age, cannot tolerate opioids for pain relief	62	56.4
34	Which of the following drugs are useful for treatment of cancer pain?	62	56.4
2	Because of an underdeveloped neurological system, children under 2 years of age have decreased pain sensitivity and limited memory of painful experiences	61	55.5
16	Beyond a certain dosage of morphine increases in dosage will NOT provide increased pain relief	60	54.5
33	The most likely explanation for why a child/adolescent with pain would request increased doses of pain medication is	59	53.6
9	Respiratory depression rarely occurs in children/adolescents who have been receiving opioids over a period of months	57	51.8
10	Acetaminophen 650 mg PO is approximately equal in analgesic effect to codeine 32 mg PO	53	48.2
20	Based on one’s religious beliefs a child/adolescent may think that pain and suffering is necessary	53	48.2
25	In order to be effective, heat and cold should be applied directly to the painful area	52	47.3
11	The World Health Organization (WHO) pain ladder suggests using single analgesic agents rather than combining classes of drugs (i.e., combining an opioid with a non-steroidal agent)	48	43.6
6	Ibuprofen and other non-steroidal anti-inflammatory agents are not effective analgesics for bone pain caused by metastases	43	39.1
23	The child/adolescent should be advised to use non-drug techniques alone rather than concurrently with pain medications	43	39.1
3	If the infant/child/adolescent can be distracted from his/her pain this usually means that he/she is not experiencing a high level of pain	42	38.2
19	Children, less than 8 years, cannot reliably report pain intensity and; therefore, the nurse should rely on the parents’ assessment of the child's pain intensity	40	36.4
36	Which of the following describes the best approach for cultural considerations in caring for child/adolescent in pain	38	34.5
21	Anxiolytics, sedatives, and barbiturates are appropriate medications for the relief of pain during painful procedures	33	30.0
29	Which of the following IV doses of morphine administered would be equivalent to 15 mg of oral morphine	31	28.2
12	The usual duration of analgesia of morphine IV is 4–5 h	28	25.5
26	The recommended route of administration of opioid analgesics to children with prolonged cancer-related pain is	28	25.5
13	Research shows that promethazine (Phenergan(R)) is a reliable potentiator of opioid analgesics	27	24.5
15	Adolescents with a history of substance abuse should not be given opioids for pain because they are at high risk for repeated addiction	25	22.7
35	The most accurate judge of the intensity of the child’s/adolescent’s pain is	25	22.7
24	Giving children/adolescents sterile water by injection (placebo) is often a useful test to determine if the pain is real	23	20.9
31	A child with chronic cancer pain has been receiving daily opioid analgesics for 2 months. The doses increased during this time period. Yesterday the child was receiving morphine 20 mg/hour intravenously. Today he/she has been receiving 25 mg/hour intravenously for 3 h. The likelihood of the child developing clinically significant respiratory depression is	22	20.0
40b	Check the action you will take at this time	21	19.1
1	Observable changes in vital signs must be relied upon to verify a child’s/adolescent’s statement that he/she has severe pain	14	12.7
4	Infants/children/adolescents may sleep in spite of severe pain	14	12.7
37	What do you think is the percentage of patients who over report the amount of pain they have? Circle the correct answer	12	10.9
38	Narcotic/opioid addiction is defined as psychological dependence accompanied by overwhelming concern with obtaining and using narcotics for psychic effect, not for medical reasons. It may occur with or without the physiological changes of tolerance to analgesia and physical dependence (withdrawal). Using this definition, how likely is it that opioid addiction will occur as a result if treating pain with opioid analgesics? Circle the number closest to what you consider the correct answer	7	6.4
7	Non-drug interventions (e.g., heat, music, imagery, etc.) are very effective for mild–moderate pain control, but are rarely helpful for more severe pain	6	5.5
39b	Check the action you will take at this time (regarding correct dose of morphine to administer)	5	4.5
40a	On the patient’s record you must mark his pain on the scale below. Circle the number that represents your assessment of Andrew’s pain	2	1.8
39a	On the patient's record you must mark his pain on the scale below. Circle the number that represents your assessment of Andrew’s pain	0	0.0

### Univariate analyses

There were no statistically significant relationships between qualification, age, years of experience, or test scores. There was also no main effect for department worked in and test scores. See Table 1. Given an absence of significant univariate tests, multivariate analyses were not conducted.

## Discussion

This study was designed to examine knowledge and attitudes regarding pain assessment and management among nurses at a tertiary children’s hospital in Nepal. Findings revealed notable gaps in nurse’s knowledge and erroneous attitudes towards pain assessment and management. No nurses met the proposed pass level of 80%, which is considered indicative of the ability to work safely and effectively in managing paediatric pain. Instead, the mean score was 38% with a range of 14% to 56% and was not influenced by years of experience, academic qualification, or hospital department worked in. The mean scores found in this study are similar to other studies undertaken with registered nurses in paediatric settings in low resource settings. For example, mean scores of registered nurses were 45% in Saudi Arabia (Alotaibi et al., 2019), 38% in Turkey (Ekim and Ocakcı, 2013), 26% in Mongolia (Lunsford, 2015), and 17% in India (Dongara et al., 2015). Work among nursing students has produced similar findings, with nursing students in Mexico producing mean scores of 40% (Ortiz et al., 2015), and final year paediatric nursing students in Ghana having mean scores of 44% (Amponsah et al., 2020a). Studies examining knowledge and attitudes of nurses in non-paediatric settings show similar findings (Shakya et al., 2020). For example, a study of 4668 registered nurses working in 48 hospitals in China reported mean scores of 40%, with no respondents scoring over 80% (Ou et al., 2021). Overall, findings indicate that deficits in pain knowledge and attitudes are universal, and therefore an important area for further education and practice development.

Findings in this study related to knowledge and the subjective nature of pain, the biopsychosocial model of pain, and the risks associated with opioid medications are routinely answered incorrectly, in line with prior work showing that patient scenarios (items 39a, 39b, 40a, 40b), the probability of narcotic addiction (item 38), and the value of non-pharmacological interventions (item 7) are consistently among the top five incorrectly answered items (Alotaibi et al., 2019, Amponsah et al., 2020a, Ortiz et al., 2015, Smeland et al., 2018).

Conversely, in this study and other studies items pertaining to pharmacological management of acute and procedural pain tend to be answered well. Specifically, items relating to the need to adjust analgesic medication according to the patient’s response (item 22), the importance of providing adequate analgesics initially when repeated procedures are planned (item 8), and that post-surgical analgesics should initially be given on a fixed schedule (item 30) (Alotaibi et al., 2019, Amponsah et al., 2020a, Ortiz et al., 2015, Smeland et al., 2018) are often among the top five correctly answered items. Of concern, in this study approximately 40% of nurses thought that children should endure as much pain as possible before using pain relief (item 18), 45% thought that children under 2 years of age are less sensitive to pain, and 77% did not think that a child/adolescent was the most accurate judge of their pain (item 35).

### Strengths

The strength of this study is that it provides the first examination of the pain knowledge and attitudes among paediatric nurses in Nepal; and provides results that will be useful in benchmarking current practice. The findings of this study provide a baseline to measure future improvements in nurses’ knowledge, attitude and practices in assessing and managing children’s pain. This study also provides evidence for recommendations for clinicians, educators and policy makers regarding the need for further professional development and training around children’s pain assessment and management. To the best of our knowledge, this study is the first of its kind in Nepal to explore nurses’ knowledge and attitude regarding children’s pain assessment and management. Hence, the findings of this study can contribute to design and implementation of a socio-culturally appropriate pain education programme for paediatric nurses working in Nepal and other low-income countries.

### Limitations

First, because it is a cross-sectional study based in a single hospital, findings may not represent pain knowledge and attitudes among nurses in Nepal more broadly. However, given that this is the third study to show suboptimal knowledge and attitudes in Nepal it seems likely that deficits exist (Shakya et al., 2020, Shakya and Shakya, 2016). Second, the present study has relatively small sample size, and power analyses were not conducted meaning that the present sample may be insufficient to detect between group differences in pain knowledge and attitudes. However, because all nurses were invited to take part, the findings do represent the full population of paediatric nurses at this hospital. Third, the PNKAS does not comprehensively assess the breadth of current recommended pain curricula, particularly with regard to the psychosocial aspects of pain management.

## Recommendations

Prior work suggests that there is currently an over emphasis on the biomedical management of pain in Nepal (Sharma et al., 2019). Therefore, ensuring that nurses receive sufficient education regarding the biopsychosocial model of pain, and paying attention to the psychosocial and cultural aspect of pain that are specifically relevant to Nepalese context is essential (Sharma et al., 2018). Research shows that nurse’s knowledge and attitudes towards pain management are influenced by the cultural context of how pain is viewed, therefore understanding the specific drivers and barriers to nurses’ management of children’s pain in Nepal is needed in order to design nursing education programs that are specific to local socio-cultural context. Further research including a STEEP (Social/Cultural, Technology, Environment, Economics, and Politics) and SWOT (Strengths, Weakness, Opportunities, Threats) analysis would provide a good foundation for planning education programs for nurses, and co-designing education programs with nurses would ensure that different experiences, competencies, and perspectives are included to bridge any knowledge translation gaps between theory and clinical practice (Elbers et al., 2021).

The findings of this study indicate that both undergraduate education and professional development in pain mechanisms, assessment, and management are urgently needed. Prior work shows that brief interventions can be effective, with paediatric nurses in Taiwan reporting increased confidence in providing pain management for children in palliative care after a 5-hour training course on pain management (Chen et al., 2017). Additionally, mean scores of nurses at a children’s hospital in Mongolia increased from 26.4% to 47.8% following a two-and-a-half-hour pain management training session (Lunsford, 2015), and scores among nurses in Mexico improved from 44% to 56% following a 4-hour education programme (Huth et al., 2010). A systematic review of education programs in low-middle income countries suggests using multiple modalities, using low-dose/high dose strategies, using peer or train the trainer models, focus on empowerment for nurses and integrating the local cultural context (Azad et al., 2020). Alongside professional development, pain education needs to become a core component of undergraduate training (Barr et al., 2006). Although many professional development training programmes are available, the Essential Pain Management (EPM) programme represents one possibility (Marun et al., 2020).

One advantage of the EPM programme is that it was designed to encourage early handover of teaching to local instructors, which has been found to improve sustainability by utilising local expertise. Once clinicians have attended the programme, they can undergo training to deliver EPM themselves. If registered nurses have the skills and resources to deliver pain education via the EPM programme, this may represent an avenue for improving pain education in undergraduate settings.

Despite apparent benefits of the EMP programme, current low scores across multiple contexts in Nepal suggest that either (a) nurses may encounter barriers to attending, or (b) the programme may not provide adequate knowledge or be sufficient to change attitudes. Hence, further work is needed to ascertain the efficacy of this programme, and/or improve access and attendance rates among nurses in Nepal. In addition, alongside pain education, research shows that nurse’s knowledge and attitudes towards pain management are influenced by the availability of resources for adequate pain management (Walters et al., 2018). Known barriers to effective pain management include limited access to pain medications, and where countries lack access to universal health care (such as Nepal) paying for medication can be a challenge for caregivers (Matula et al., 2018). Other barriers to delivery of evidence-based care include unhelpful parental beliefs, insufficient prescription of analgesics by physicians (Amponsah et al., 2020b, Twycross, 2013), and a lack of adequately validated pain assessment tools (Sharma et al., 2016, 2019). Conversely, facilitators to effective pain management include parental participation in care, trusting and respectful relationships between nurses and children, and adequate nurse–patient ratios (Alotaibi et al., 2018). Therefore, in addition to providing pain education for nurses, improving paediatric pain management in low- and middle-income countries requires education of families, advocacy for effective pain management, improving treatment availability (Morriss and Roques, 2018), and the development of non-pharmacological pain management programmes that focus on low-cost strategies, such as distraction, hypnosis, or relaxation techniques (Walters et al., 2018).

Finally, although undergraduate pain education and continued professional development are key to improving knowledge and attitudes among nurses, knowledge does not always translate into clinical practice (Stevens et al., 2014). Despite multiple barriers, supporting nurses to deliver evidence-based care represents a pragmatic starting point. Qualitative work is needed to generate an in-depth understanding of the barriers and facilitators to effective pain assessment and management within this particular setting, and to co-design the most appropriate program for professional development.

## Conclusion

The present study is the first to document knowledge and attitudes of the total population of paediatric nurses in a tertiary children’s hospital in Nepal. Findings are consistent with international research showing that knowledge and attitudes of paediatric nurses are suboptimal and inconsistent with evidence-based practice, no nurses in this population met the proposed threshold for safe practice (scores ≥80%). Findings demonstrate that pain education is urgently needed at both the undergraduate and post-registration level to support nurses to deliver evidence-based care and to improve health outcomes for children and support their rights to not experience pain needlessly. Future work could identify the most appropriate way to co-design and deliver effective pain education and measure the degree to which pain education opportunities improves pain knowledge and attitudes among paediatric nurses in Nepal.

## Relevance to clinical practice

Optimal pain assessment and management in children has beneficial health, psychosocial, and economic outcomes. Medical training facilities and hospitals must prioritize pain education to meet international pain management guidelines; and enable nurses to deliver evidence-informed care for accurate assessment and effective management of pain in paediatric practice.
